# Phytase supplementation in sorghum-based diet enhances nutrient digestibility, energy utilization, and antioxidant status of Campbell ducks

**DOI:** 10.1016/j.psj.2025.106198

**Published:** 2025-12-05

**Authors:** Z. Li, F. Raziq, M.T. Khan, S. Ali, A. Ullah, I. Ahmed, A.A. Alfaleh, M.A. Albalawi, A.E. Ahmed, N. Al-Hoshani, H. Yuan

**Affiliations:** a Qujing Normal University, College of Biological Resource and Food Engineering 655011, Yunnan, China; b Department of Poultry Production, Faculty of Animal Production and Technology, University of Veterinary and Animal Sciences, Lahore 54000, Pakistan; c Department of Poultry Science, Faculty of Animal Production and Technology, Cholistan University of Veterinary and Animal Sciences, Bahawalpur 63100, Pakistan; d Central Diagnostic Laboratory, Cholistan University of Veterinary and Animal Sciences, Bahawalpur 63100, Pakistan; e Department of Poultry Science, Faculty of Animal Husbandry and Veterinary Sciences, The University of Agriculture, Peshawar 25130, Pakistan; f Institute of Microbiology, Faculty of Veterinary Sciences, University of Veterinary and Animal Sciences, Lahore 54000, Pakistan; g Department of Animal Nutrition, Faculty of Veterinary and Animal Sciences, The Islamia University of Bahawalpur, Bahawalpur 63100, Pakistan; h Food and Nutrition Department, Faculty of Human Sciences and Design, King Abdulaziz University, Jeddah, Saudi Arabia; i Department of Chemistry, Alwajh College, University of Tabuk, Saudi Arabia; j Department of Biology, College of Science, King Khalid University, Abha 61413, Saudi Arabia; k Prince Sultan Bin Abdelaziz for Environmental Research and Natural Resources Sustainability Center, King Khalid University, Abha 61421, Saudi Arabia; l Department of Biology, College of Science, Princess Nourah bint Abdulrahman University, Riyadh 11671, Saudi Arabia

**Keywords:** Phytase supplementation, Sorghum varieties, Campbell ducks, Nutrient digestibility, Antioxidant status

## Abstract

Sorghum is an energy-dense cereal grain with substantial potential for poultry nutrition. However, its practical use is often restricted due to high phytate content, which reduces nutrient digestibility and mineral bioavailability. This study evaluated the effects of phytase supplementation on nutrient digestibility, mineral retention, energy utilization, and antioxidant status in Campbell ducks (*Anas platyrhynchos*) fed red and white sorghum-based diets. A total of forty-eight adult ducks (1.65 ± 0.05 kg) were randomly assigned to 12 metabolic cages (four ducks per cage) and subjected to a 10-day feeding trial, which included 4 days of acclimation followed by 6 days of fecal collection. The experimental treatments consisted of red or white sorghum diets with or without supplemental phytase (500 FTU/kg). Phytase supplementation significantly (*P<* 0.05) improved apparent dry matter digestibility, crude protein digestibility, and apparent metabolizable energy in ducks fed sorghum-based diets compared to the non-supplemented control. Additionally, calcium and phosphorus retention were significantly enhanced in the phytase-supplemented groups, with the highest retention observed in ducks receiving the red sorghum plus phytase diet. Antioxidant status also improved, as indicated by significant (*P<* 0.05) increases in superoxide dismutase, glutathione peroxidase, and catalase activities, along with reduced malondialdehyde concentrations, demonstrating lower lipid peroxidation and improved oxidative stability in phytase-fed ducks. In conclusion, phytase supplementation effectively mitigated the anti-nutritional effects of phytate, improved nutrient digestibility, enhanced mineral retention, and strengthened antioxidant defense mechanisms, with the most significant improvements seen in ducks fed red sorghum-based diets.

## Introduction

The global poultry industry, including the rapidly growing waterfowl sector, faces increasing pressure to adopt efficient, cost-effective, and sustainable feeding strategies to meet the rising global demand for high-quality animal protein. Ducks (*Anas platyrhynchos*), an economically important avian species, require energy-dense and nutritionally balanced diets to achieve optimal growth, feed efficiency, and meat quality. In this context, sorghum (*Sorghum bicolor* L.) has gained considerable attention as a promising alternative to maize due to its superior drought tolerance, adaptability to marginal environments, and low input requirements, making it particularly suitable for arid and semi-arid production systems (Mehany et al., 2023). Despite these advantages, the nutritional value of sorghum in monogastric diets is limited by its high phytate (inositol hexakisphosphate) content. Phytate forms insoluble complexes with essential minerals, such as calcium (Ca), zinc (Zn), and magnesium (Mg), which reduces their bioavailability and impairs nutrient digestibility (Selle et al., 2023). This reduced digestibility of phytate-bound nutrients can lead to suboptimal growth performance, compromised bone mineralization, and increased nutrient losses, contributing to environmental phosphorus (P) excretion (Liu et al., 2020). Since ducks have minimal endogenous phytase activity, the supplementation of exogenous phytase has emerged as an effective nutritional strategy to enhance nutrient utilization and improve the sustainability of sorghum-based feeding systems (Dersjant-Li et al., 2022).

Exogenous phytase hydrolyzes phytate, releasing bound P, Ca, amino acids (AAs), and metabolizable energy, thereby enhancing growth performance, feed efficiency, and nutrient digestibility (Hernandez et al., 2022; Rodehutscord et al., 2022). Extensive research on broilers and layers has shown that phytase supplementation improves body weight gain (BWG), feed conversion ratio (FCR), and bone mineralization while reducing the need for inorganic P supplementation (Bougouin et al., 2014; Wu et al., 2021). Although research on ducks is comparatively limited, existing studies suggest that phytase can similarly enhance nutrient digestibility, carcass characteristics, and overall productivity in waterfowl (Jiang et al., 2020). Since sorghum contains higher levels of phytate and lower intrinsic phytase activity than maize, ducks on sorghum-based diets are expected to respond particularly well to phytase inclusion (Yu et al., 2023). Among the commonly applied supplementation levels, 500 FTU/kg is widely regarded as both biologically effective and economically practical, as it consistently improves phytate hydrolysis, AA availability, mineral retention, and energy utilization without disrupting nutrient balance or unnecessarily increasing feed costs (Jones et al., 2009; Truong et al., 2017).

Despite the extensive evidence available for chickens, limited information exists regarding the effects of phytase in Campbell ducks—a widely farmed dual-purpose breed known for its high feed efficiency, adaptability, and commercial relevance (Nuamah et al., 2024). Ducks differ from chickens in their digestive physiology, feeding behavior, and nutrient metabolism, highlighting the need for species-specific evaluations of enzyme supplementation strategies (Park and Carey, 2019). Additionally, little is known about the comparative effects of phytase on nutrient digestibility, mineral retention, and antioxidant status in ducks fed red versus white sorghum varieties. Therefore, this study aims to evaluate the impact of dietary phytase supplementation on growth performance, nutrient digestibility, mineral retention, carcass traits, and antioxidant status in Campbell ducks fed red and white sorghum-based diets.

## Materials and methods

### Animal ethics approval

All procedures and experimental protocols involving birds were conducted in accordance with the guidelines and regulations set forth by the Animal Ethics Committee of the Faculty of Animal Husbandry and Veterinary Sciences at The University of Agriculture, Peshawar (UAP), Pakistan. Ethical approval for the study was obtained prior to the commencement of the experiment (Certificate No. 455/PS/UAP, dated 05/02/2022).

### Experimental site and birds

The research was conducted at the Poultry Research Complex, UAP, Pakistan, in May 2022. A total of 48 adult Campbell ducks were maintained under standard management and husbandry conditions at the university farm. Throughout the experimental period, representative samples of feed, feces, and liver tissue were collected for subsequent laboratory analyses. Feed and fecal samples were analyzed for proximate composition [dry matter (DM), crude protein (CP), ether extract (EE), crude fiber (CF), and crude ash (CA)], mineral content (Ca and P), and energy values at the Pakistan Council of Scientific and Industrial Research, Peshawar. Liver samples were submitted to the Veterinary Research Institute, Peshawar, for the determination of antioxidant enzyme activities, including superoxide dismutase (SOD), glutathione peroxidase (GPx), and catalase (CAT), as well as lipid peroxidation levels (malondialdehyde; MDA). These biochemical assessments were used to evaluate the oxidative status of the ducks in response to dietary treatments.

### Digestibility trial

In this digestibility trial, a total of 48 adult Campbell ducks (*Anas platyrhynchos*) were obtained from the Poultry Research Complex at the Faculty of Animal Husbandry and Veterinary Sciences, UAP, Pakistan. The birds were moved from the main flock housing facility to 12 metabolic cages with four birds per cage, to facilitate precise measurement of feed intake (FI) and complete fecal collection. The experiment lasted for 10 days, including a 4-day acclimation period followed by 6 days of fecal collection.

Each metabolic cage measured 75 cm (L) × 55 cm (W) × 60 cm (H), providing a total usable floor area of 2.16 m² (0.54 m² per bird), which is consistent with recommended space allowances for medium-sized waterfowl in semi-intensive research settings (Mehany et al., 2023). The cages featured a slightly sloped wire-mesh floor (5–7°) to facilitate feces separation and were equipped with removable plastic trays to ensure hygienic and uncontaminated excreta collection. Ducks were housed individually during the collection phase to prevent cross-contamination and to ensure accurate quantification of nutrient intake and excretion. The experimental room maintained a controlled temperature of 22–25°C and relative humidity of 55–65 %, monitored using a wet- and dry-bulb hygrometer (Mason’s Type, London SW19 3UU, England). A 16:8 h light–dark cycle was implemented throughout the trial. Feed and water were offered ad libitum in spill-proof containers to minimize wastage and contamination, following NRC (1994) recommendations (Table 1, Table 2).

**Table 1:** Composition of the sorghum based diets.

Ingredients (%)	Red sorghum	White sorghum
Sorghum	45.10	45.12
Wheat bran	10.00	10.00
Rice polish	12.00	12.00
Soybean meal (44 %)	13.90	13.86
Sunflower meal	6.00	6.00
Fish meal	4.00	4.00
Limestone	4.46	4.48
Dicalcium phosphate	1.04	1.06
Salt (NaCl)	0.30	0.30
Vitamin–mineral premix	0.25	0.25
DL-Methionine	0.10	0.10
L-Lysine	0.05	0.05
Total	100.00	100.00
Nutrient composition (calculated)		
Metabolizable energy (kcal/kg)	2815	2820
Crude protein	17.5	17.5
Calcium	0.35	0.35
Available phosphorus	0.45	0.46
Lysine	0.75	0.75
Methionine	0.35	0.34

**Table 2:** Nutrient profile of two different varieties of sorghum fed to adult Campbell ducks (as fed).

Nutrients (%)	Sorghum variety	
	Red type	White type
Dry matter%	91.82	91.88
Organic matter%	94.65	95.84
Crude protein%	11.48	9.97
Starch%	73.89	76.34
Crude fat%	3.23	2.91
Crude fiber%	2.13	2.32
Total Phosphorus%	0.37	0.32
Calcium%	0.05	0.04
Phytase%	0.85	0.75
Crude ash%	2.94	2.23
Metabolizable energy (kcal/kg)	3700	3750

During the 6-day collection phase, daily feed intake and excreta output were recorded. Feces were collected each morning at 06:00 h, pooled by cage, and immediately stored at –20°C until analysis. Composite fecal samples were subsequently analyzed for DM, CP, EE, CF, and CA, following standard AOAC (2019) procedures.

### Inclusion of Phytase in Diet

Phytase enzyme (Natuphos® E, BASF, Germany) was obtained from an authorized local distributor, Ghazi Brothers (Pakistan), and added to the experimental diets at the manufacturer-recommended inclusion rate of 500 FTU/kg. The enzyme was thoroughly mixed into the diets to ensure uniform distribution and maintain its catalytic activity. The study employed a 2 × 2 factorial design, consisting of two sorghum varieties (red and white), each with or without phytase supplementation. This resulted in four dietary treatments: RS₀ (red sorghum without phytase), RS₅₀₀ (red sorghum with 500 FTU/kg phytase), WS₀ (white sorghum without phytase), and WS₅₀₀ (white sorghum with 500 FTU/kg phytase). All diets were formulated to be i*so-nitrogenous* and *iso-caloric*, meeting or exceeding the nutrient requirements for adult ducks as specified by the NRC (1994). Phytase incorporation and handling were conducted under controlled temperature and humidity to maintain enzyme stability, ensuring consistent phytate hydrolysis and P release during digestion, in accordance with standard recommendations (Cowieson et al., 2019).

### Data collection

#### Growth performance

The growth performance of Campbell ducks was assessed across all replicates by recording weekly body weights and feed consumption throughout the experiment. Individual birds were weighed using a digital scale (Adam Equipment LBK, UK) to determine BWG. Daily feed offered and residual feed were recorded to calculate FI. The FCR was calculated as the ratio of FI to BWG.

#### Carcass characteristics

At the end of the feeding trial, two ducks per replicate were randomly selected and fasted for 12 hours, with free access to drinking water, to facilitate gut clearance. The birds were humanely euthanized by cervical dislocation in accordance with the approved guidelines of the Animal Ethics Committee at UAP, Pakistan. Immediately after euthanasia, the ducks were defeathered, eviscerated, and their carcasses weighed to determine the dressing percentage (DP), calculated as the ratio of eviscerated carcass weight to live body weight. The breast muscles (pectoralis major and minor) were carefully excised and weighed to determine breast yield (BY), expressed as a proportion of the carcass weight. All measurements were performed immediately post-processing using a calibrated digital scale (SF-400A, Zhongshan Camry Electronic Co., Ltd, China), following standardized meat evaluation protocols (Jin et al., 2021).

#### Nutrient digestibility

Representative feed and fecal samples (approximately 200 g each) were collected from adult Campbell ducks in each replicate and analyzed for proximate composition and mineral content following the procedures described by Aletan and Kwazo (2019). All analyses were performed in triplicate to ensure accuracy and reproducibility. Dry matter was determined by oven drying 2–5 g of sample at 105 °C for 16 hours until a constant weight was achieved (AOAC 934.01). Ether extract was measured using a Soxhlet apparatus with petroleum ether (boiling point 40–60 °C) for 6–8 hours in accordance with AOAC (2019) Method 920.39. Calcium content was determined by complexometric titration with ethylenediaminetetraacetic acid (EDTA) after dry ashing samples at 550 °C for 6 hours, using murexide as the endpoint indicator (AOAC 927.02). Phosphorus was determined colorimetrically following acid digestion, using the vanadomolybdate method (AOAC 965.17). The absorbance of the resulting yellow phosphovanadomolybdate complex was measured at 430 nm using a UV–Visible spectrophotometer (Shimadzu UV-1800, Japan).

#### Apparent Nutrient Digestibility

Apparent nutrient digestibility coefficients for DM, CP, EE, CF, and CA were determined using a modified total fecal collection method for waterfowl metabolism studies (Hill and Anderson, 1958; Sibbald, 1976). During the 6-day collection phase, daily feed intake and fecal output were recorded for each replicate. Fecal samples were then oven-dried, ground, and analyzed for proximate composition according to AOAC (2019) procedures. Apparent digestibility was calculated using the following formula:

$$\text{Apparent}\;\text{digestibility}\;\left(\%\right)=\frac{Nutrient\;intake-Nutrient\;output\;in\;feces}{Nutrient\;intake}\times \;100$$

Nutrient intake and fecal nutrient output were calculated based on the actual feed consumed and the total excreta collected per replicate, following the method described by Adeola and Cowieson (2011).

#### Apparent metabolizable energy

Gross energy (GE) refers to the total chemical energy found in feed ingredients and serves as a key indicator of the dietary energy available to poultry, including Campbell ducks. In this study, GE was estimated using a predictive equation based on the proximate composition of the experimental diets, following the method described by Adeola and Cowieson (2011). This indirect approach provides a reliable and widely accepted estimate of dietary energy content based on macronutrient profiles and acts as an effective alternative to direct measurement through bomb calorimetry.

#### Antioxidant status

At the end of the trial, two ducks from each replicate were randomly selected and humanely euthanized through cervical dislocation followed by exsanguination, in accordance with the international poultry welfare standards established by the World Organization for Animal Health (OIE, 2021) and the American Veterinary Medical Association (AVMA, 2020), and as approved by the Animal Ethics Committee of the Faculty of Animal Husbandry and Veterinary Sciences, UAP, Pakistan. Immediately after euthanasia, approximately 5 g of liver tissue were aseptically collected from each bird, rinsed in ice-cold saline, and snap-frozen in liquid nitrogen to prevent oxidative or enzymatic degradation. The samples were stored at −80°C until biochemical analysis.

For antioxidant enzyme assays, liver tissues were homogenized (1:10, w/v) in ice-cold phosphate-buffered saline (pH 7.4) using a chilled homogenizer. The homogenate was then centrifuged at 10,000 × *g* for 15 minutes at 4°C, and the resulting clear supernatant was used for enzymatic analyses. Superoxide dismutase activity was determined by its capacity to inhibit the photoreduction of nitroblue tetrazolium (Marklund and Marklund, 1974). Glutathione peroxidase activity was measured using a coupled enzyme system by monitoring the oxidation of NADPH at 340 nm (Paglia and Valentine, 1967). Catalase activity was assessed by measuring the decomposition rate of hydrogen peroxide (H₂O₂) at 240 nm (Aebi, 1984). Enzyme activities were expressed as U/mg protein, with total protein concentration determined using the Bradford method (Bradford, 1976). All assays were performed in triplicate using a UV–Visible spectrophotometer (UV-1800, Shimadzu, Japan) to ensure analytical precision and reproducibility.

### Statistical analysis

The experiment was conducted using a 2 × 2 factorial arrangement within a completely randomized design, evaluating two factors at two levels each. All collected data were compiled and organized in Microsoft Excel (Version 2007) and then analyzed using the General Linear Model (GLM) procedure of SAS software (SAS Institute Inc., Cary, NC, 2002–2003), following the statistical procedures outlined by Meyer et al. (2009).

The following statistical model was used:

$$Y_{ijk}=\mu +S_{i}+P_{j}+{\left(S\times P\right)}_{ij}+\varepsilon ijk,$$

Where Y_ijk_ = Observed dependent variable; μ = Overall mean; S_i_ = Effect of sorghum variety; P_j_ = Effect of phytase enzyme; (*S* × *P*)_ij_ = Interaction in sorghum variety and phytase enzyme; and ε_ijk_ = Residual error.

## Results

### Growth performance and carcass characteristics

Phytase supplementation significantly enhanced (*P <* 0.05) the growth performance and carcass characteristics of Campbell ducks fed sorghum-based diets (Table 3). Ducks receiving phytase (RS₅₀₀ and WS₅₀₀) showed higher FI, greater BWG, and improved FCR compared to their non-supplemented counterparts. Carcass traits were also enhanced, with phytase increasing breast muscle percentage and dressing percentage in both sorghum varieties. Although ducks fed red sorghum generally performed slightly better than those fed white sorghum, phytase consistently improved all measured parameters regardless of sorghum type.

**Table 3:** Effect of phytase supplementation in sorghum-based diet on growth performance and carcass characteristics of Campbell ducks^1^.

Treatments^3^		Parameters^2^				
		FI (g)	BWG (g)	FCR	BM (%)	DP (%)
Sorghum	Phytase					
RS	RS₅₀₀	2776a	1525a	1.82b	24.18a	72.45a
	RS₀	2704b	1421b	1.90a	22.45b	69.82b
WS	WS₅₀₀	2763a	1491a	1.85b	23.12a	71.35a
	WS₀	2690b	1388b	1.94a	21.78b	68.90b
SEM		12.264	9.671	0.023	2.146	5.821
P-value						
Sorghum		0.032	0.041	0.022	0.046	0.049
Phytase		0.015	0.034	0.031	0.051	0.033

1 Data are means + SE of 3 replicates (n = 3) with four birds per replicate.

2 FI: feed intake, BWG: body weight gain, FCR: feed conversion ratio, BM: breast muscle, DP: dressing percentage.

3 RS: red sorghum, WS: white sorghum, RS₅₀₀: Red sorghum diet with 500 FTU phytase per kg, RS₀: Red sorghum diet without phytase, WS₅₀₀: white sorghum diet with 500 FTU phytase per kg, WS₀: white sorghum diet without phytase, SEM: standard error of the mean. a-bMeans within a column lacking a common superscript differ (P < 0.05).

### Nutrient digestibility

Phytase supplementation significantly enhanced nutrient digestibility in Campbell ducks fed sorghum-based diets (*P <* 0.05; Table 4). Ducks receiving phytase (RS₅₀₀ and WS₅₀₀) exhibited higher DM and OM digestibility, along with better nitrogen retention and overall nutrient digestibility compared to their respective non-supplemented groups. The digestibility of EE, CF, and crystallized fiber (CRF) was also improved. Furthermore, phytase significantly enhanced P and phytate digestibility, resulting in higher apparent metabolizable energy (AME) values for both sorghum varieties. Although red sorghum exhibited slightly superior digestibility than white sorghum, phytase consistently improved all evaluated parameters, regardless of sorghum type.

**Table 4:** Effect of phytase supplementation in sorghum-based diet on nutrient digestibility of Campbell ducks.

Treatments^2^		Parameters^1^									
		DM	OM	NR	ND	EE	CF	CRF	P	PT	AME
Sorghum	Phytase										
RS	RS₅₀₀	0.83.3a	0.854a	0.633a	0.732a	0.632a	0.662a	0.629a	0.589a	0.362a	14.62a
	RS₀	0.798b	0.834b	0.599b	0.724b	0.599b	0.644b	0.599b	0.53b	0.224b	13.8b
WS	WS₅₀₀	0.799a	0.861a	0.599a	0.731a	0.599a	0.652a	0.631a	0.579a	0.352a	13.9a
	WS₀	0.786b	0.843b	0.596b	0.723b	0.579b	0.638b	0.599b	0.569b	0.236b	13.3b
SEM		0.049	0.057	0.077	0.042	0.024	0.33	0.041	0.052	0.037	0.037
P-value											
Sorghum		0.03	0.04	0.03	0.13	0.03	0.07	0.08	0.07	0.08	0.08
Phytase		0.04	0.03	0.02	0.08	0.02	0.02	0.04	0.01	0.01	0.03

1 DM: dry matter, OM: organic matter, NR: nitrogen retention, ND: nutrient digestibility, EE: ether extract, CF: crude fiber, CRF: crystallized fiber digestibility, P: phosphorous, PT: phytate, AME: apparent metabolizable energy.

2 RS: red sorghum, WS: white sorghum, RS₅₀₀: Red sorghum diet with 500 FTU phytase per kg, RS₀: Red sorghum diet without phytase, WS₅₀₀: white sorghum diet with 500 FTU phytase per kg, WS₀: white sorghum diet without phytase, SEM: standard error of the mean. a-bMeans within a column lacking a common superscript differ (P < 0.05).

### Antioxidant Status

Phytase supplementation significantly enhanced the antioxidant status of Campbell ducks fed sorghum-based diets (*P <* 0.05; Table 5). Ducks receiving phytase (RS₅₀₀ and WS₅₀₀) exhibited higher activities of SOD, GPx, and CAT compared to non-supplemented groups. Additionally, MDA levels were significantly reduced, indicating lower lipid peroxidation and oxidative stress. Although red sorghum tended to support slightly greater antioxidant enzyme activities and lower MDA levels than white sorghum, phytase consistently improved all measured parameters, regardless of sorghum variety.

**Table 5:** Effect of phytase supplementation in sorghum-based diet on antioxidant status of Campbell ducks.

Treatments^2^		Parameters^1^			
		SOD (U/mg)	GPx (U/mg)	CAT (U/mg)	MDA (nmol/mg)
Sorghum	Phytase				
RS	RS₅₀₀	62.14a	38.27a	51.33a	1.92b
	RS₀	53.44b	32.80b	45.02b	2.86a
WS	WS₅₀₀	59.11a	36.52a	48.91a	2.03b
	WS₀	50.08b	90.94b	42.77b	2.74a
SEM		3.291	3.024	2.016	0.006
P-value					
Sorghum		0.032	0.014	0.48	0.039
Phytase		<0.001	0.038	0.022	0.011

1 SOD: superoxide dismutase, GPx: glutathione peroxidase, CAT: catalase, MDA: malondialdehyde.

2 RS: red sorghum, WS: white sorghum, RS₅₀₀: Red sorghum diet with 500 FTU phytase per kg, RS₀: Red sorghum diet without phytase, WS₅₀₀: white sorghum diet with 500 FTU phytase per kg, WS₀: white sorghum diet without phytase, SEM: standard error of the mean. a-bMeans within a column lacking a common superscript differ (P < 0.05).

## Discussion

Sorghum is increasingly recognized as a climate-resilient and drought-tolerant cereal crop, offering a sustainable alternative to maize in poultry nutrition due to its high energy density and adaptability to marginal agro-ecological conditions (Mehany et al., 2023). For meat-type ducks, such as the Campbell breed (*Anas platyrhynchos*), sorghum represents a cost-effective feed ingredient. However, its nutritional value in monogastric diets is limited by the presence of anti-nutritional factors, particularly phytic acid. Phytic acid strongly chelates essential minerals and proteins, forming insoluble complexes that compromise their digestibility and absorption (Cowieson and Parsons, 2024). Ducks, like other monogastric species, produce insufficient endogenous phytase to hydrolyze dietary phytate, making the inclusion of exogenous microbial phytase essential. Phytase is widely used to enhance P release, improve mineral and protein digestibility, and mitigate environmental P excretion (Dersjant-Li et al., 2015). However, the efficacy of phytase can vary among sorghum varieties due to differences in phytate content, tannin levels, and grain structure, which influence nutrient availability and digestive efficiency (Nyachoti et al., 1997). While phytase has been extensively studied in chickens, research on ducks remains limited (Jin et al., 2021). This knowledge gap underscores the need for targeted studies to optimize sorghum-based diets and improve the efficiency and sustainability of duck production.

### Nutrient profile of sorghum

Table 2 presents the chemical composition of red and white sorghum varieties fed to adult Campbell ducks, highlighting key differences in protein, starch, and mineral content. Red sorghum contained a higher CP level (11.48 %) than white sorghum (9.97 %), indicating greater potential for amino acid availability to support muscle growth (Jin et al., 2021). In contrast, white sorghum had a slightly higher starch content (76.34 % vs. 73.89 %), offering a marginal advantage as a readily available energy source (Mabelebele et al., 2015). Additionally, red sorghum exhibited slightly higher crude fat and intrinsic phytase activity, contributing to increased energy density and partial phytate degradation (Selle et al., 2023). Total P levels were higher in red sorghum (0.37 %) compared to white sorghum (0.32 %). However, most of this P remains unavailable without exogenous phytase supplementation (Nuamah et al., 2024). Additionally, red sorghum had a greater CA content, indicating a higher level of inorganic residue. These compositional differences underscore the importance of selecting appropriate sorghum genotypes for duck diets, particularly when incorporating enzyme additives to optimize nutrient release and feed conversion efficiency (Dersjant-Li et al., 2015).

### Growth performance and carcass characteristics

Phytase supplementation significantly enhanced the growth performance and carcass characteristics of Campbell ducks fed sorghum-based diets, as evidenced by higher FI, greater BWG, improved FCR, and increased breast muscle and dressing percentages. These enhancements are primarily due to the enzymatic hydrolysis of dietary phytate, which releases bound P and mitigates its anti-nutritional effects, thereby improving overall nutrient availability. The degradation of phytate also liberates AAs, proteins, and energy that were previously trapped in phytate complexes, resulting in improved digestibility and metabolic efficiency (Selle et al., 2023). Furthermore, the release of inositol during phytate breakdown may stimulate metabolic pathways that support growth and muscle development. Collectively, the improved utilization of P and energy likely facilitated more efficient nutrient partitioning toward lean tissue deposition, explaining the superior carcass traits observed in phytase-supplemented ducks.

These findings are consistent with recent poultry research showing that phytase supplementation enhances growth performance and nutrient utilization in ducks and broilers fed sorghum- or corn-based diets (Anyaegbu et al., 2021; Attia et al., 2019; Iqbal et al., 2023; Selle et al., 2023). For example, Attia et al. (2019) documented improved weight gain and feed efficiency in phytase-supplemented White Pekin ducks. Similar studies in broilers further confirm that phytase enhances growth performance, nutrient digestibility, and carcass yield, particularly in low-phosphorus or sorghum-based feeding systems (Kim et al., 2021; Mulvenna et al., 2022; Paul et al., 2025; Yu et al., 2023). Although ducks fed red sorghum exhibited slightly superior performance—likely due to its higher nutrient density and bioactive components—phytase consistently improved performance and carcass traits across all sorghum varieties. Overall, these findings reinforce phytase as an effective nutritional strategy for enhancing growth efficiency, nutrient utilization, and lean tissue deposition in sorghum-based duck diets.

### Nutrient digestibility

Phytase supplementation significantly improved nutrient digestibility in Campbell ducks fed sorghum-based diets, primarily through the enzymatic hydrolysis of dietary phytate. This process releases bound P and reduces the anti-nutritional effects of phytate, thereby enhancing the availability of essential nutrients. The degradation of phytate also liberates AAs, proteins, lipids, and energy that were previously bound within phytate–nutrient complexes. This explains the observed improvements in DM, OM, nitrogen retention, EE, CF, CRF digestibility, and apparent metabolizable energy (**AME**). Additionally, phytate hydrolysis produces myo-inositol, a bioactive compound that supports energy metabolism and mineral utilization, further promoting efficient nutrient partitioning. These mechanisms align with recent poultry studies demonstrating enhanced p digestibility, nutrient retention, and AME following phytase supplementation in cereal-based diets (Ahmad et al., 2024; de França et al., 2023; Maria et al., 2024; Munawar et al., 2022; Sasia et al., 2023; Valente Junior et al., 2024; Walk et al., 2024). For instance, phytase has been shown to increase ileal p digestibility and nutrient transporter expression in broilers (Walk et al., 2024), improve p availability and overall digestibility in poultry (Munawar et al., 2022), and elevate AME and nutrient utilization in sorghum-based and low-phosphorus diets (Ahmad et al., 2024; de França et al., 2023; Sasia et al., 2023). Additionally, improved Zn retention and higher plasma myo-inositol levels further support the metabolic benefits of phytate hydrolysis (Maria et al., 2024; Valente Junior et al., 2024). Collectively, these findings establish phytase as an effective nutritional strategy for enhancing nutrient release, digestibility, and energy utilization in sorghum-based duck diets.

### Antioxidant status

Phytase supplementation significantly enhanced the antioxidant status of Campbell ducks fed sorghum-based diets, as evidenced by increased activities of SOD, GPx, and CAT, alongside a marked reduction in MDA levels. These improvements can possibly be attributed to several complementary mechanisms. First, the enzymatic hydrolysis of dietary phytate increases the bioavailability of trace minerals such as Zn, Mn, and Se—essential cofactors required for the optimal synthesis and catalytic function of antioxidant enzymes. Enhanced mineral absorption directly strengthens the endogenous antioxidant defense system, enhancing the birds’ ability to neutralize reactive oxygen species (Derakhshan et al., 2023; Elbaz et al., 2023). Second, the breakdown of phytate improves the utilization of P, AAs, and dietary energy, resulting in more efficient cellular metabolism and a reduced intracellular oxidative burden. This improvement in metabolic efficiency decreases the production of reactive oxygen species, thereby lowering lipid peroxidation, as indicated by reduced MDA levels (Derakhshan et al., 2023; Maria et al., 2024). Third, phytate hydrolysis releases myo-inositol, a bioactive molecule that regulates mitochondrial energy metabolism, intracellular signaling pathways, and cellular redox balance. The increased availability of myo-inositol provides additional protection against oxidative stress and supports the stabilization of the overall antioxidant system (Gonzalez-Uarquin et al., 2020; Nuamah et al., 2024). Together, these mechanisms explain the simultaneous upregulation of antioxidant enzymes and reduction in lipid peroxidation observed in phytase-supplemented ducks. These findings are consistent with recent poultry research demonstrating that phytase enhances antioxidant enzyme activities, reduces oxidative damage, and improves mineral retention and metabolic efficiency in birds on cereal-based diets (Derakhshan et al., 2023; Elbaz et al., 2023; Gonzalez-Uarquin et al., 2020; Maria et al., 2024; Nuamah et al., 2024). Overall, the enhanced enzymatic antioxidant capacity and reduced oxidative damage confirm that phytase effectively mitigates oxidative stress and promotes better oxidative homeostasis in sorghum-based duck diets.

## Conclusions

The present study demonstrated that phytase supplementation significantly enhanced growth performance, carcass traits, nutrient digestibility, and antioxidant status in Campbell ducks fed sorghum-based diets. Ducks receiving phytase (RS₅₀₀ and WS₅₀₀) exhibited higher FI, greater BWG, and improved FCR, along with increased breast muscle yield and dressing percentage. Phytase also improved digestibility of protein, fat, and phosphorus, enhanced mineral retention, and increased AME. Antioxidant enzyme activities (SOD, GPx, CAT) were elevated, while MDA levels decreased, indicating reduced oxidative stress. Although red sorghum provided slightly better performance than white sorghum, phytase consistently improved all measured parameters, confirming its effectiveness as a dietary supplement for optimizing sorghum-based duck nutrition.

## CRediT authorship contribution statement

**Z. Li:** Writing – review & editing. **F. Raziq:** Formal analysis. **M.T. Khan:** Writing – review & editing. **S. Ali:** Writing – review & editing. **A. Ullah:** Writing – review & editing. **I. Ahmed:** Writing – review & editing. **A.A. Alfaleh:** Writing – review & editing. **M.A. Albalawi:** Writing – review & editing. **A.E. Ahmed:** Writing – review & editing. **N. Al-Hoshani:** Writing – review & editing. **H. Yuan:** Writing – review & editing.

## Disclosures

No conflict of interests was reported by the authors regarding the publication of this research article.
